# Dream Recall/Affect and the Hypothalamic–Pituitary–Adrenal Axis

**DOI:** 10.3390/clockssleep3030027

**Published:** 2021-07-22

**Authors:** Athanasios Tselebis, Emmanouil Zoumakis, Ioannis Ilias

**Affiliations:** 1 Department of Psychiatry, Sotiria Hospital, 115 27 Athens, Greece; atselebis@yahoo.gr; 2 First Department of Pediatrics, National and Kapodistrian University of Athens Medical School, Aghia Sophia Children’s Hospital, 115 27 Athens, Greece; zoumakis@bioacademy.gr; 3 Department of Endocrinology, Diabetes and Metabolism, Elena Venizelou Hospital, 115 21 Athens, Greece

**Keywords:** dreams, cortisol, stress, memory, sleep, HPA axis

## Abstract

In this concise review, we present an overview of research on dream recall/affect and of the hypothalamic–pituitary–adrenal (HPA) axis, discussing caveats regarding the action of hormones of the HPA axis (mainly cortisol and its free form, cortisol-binding globulin and glucocorticoid receptors). We present results of studies regarding dream recall/affect and the HPA axis under physiological (such as waking) or pathological conditions (such as in Cushing’s syndrome or stressful situations). Finally, we try to integrate the effect of the current COVID-19 situation with dream recall/affect vis-à-vis the HPA axis.

## 1. Introduction—Dream Recall

Almost all humans dream (indeed, there may be 0.5% of people who do not do so) [1]. Dreams are a series of images, thoughts and senses and are considered a specific type of experience that occurs in our brain during sleep. During the dream, there is limited control of the dream content, visual images and memory activation. Dreams tend to be related to themes, ideas, experiences, figures and objects of each person’s individual life. Dreaming and dream recall (including dreams’ affect) are human universals [2]. Most people partially recall their dreams and the ability to recall dreams varies from person to person [3,4,5]. The frequency of dream recall usually increases in childhood/adolescence, shows a plateau in adulthood and may drop slightly in old age, particularly the duration of rapid eye movement (REM) sleep [6,7,8,9]. More dreams from the REM sleep phase are recalled compared to dreams from the non-REM sleep phases [10]. Personality traits as well as situational conditions may influence dream recall [11]. Sleep/dream experts indicate that outside the sleep laboratory, dream recall may be coaxed using certain tricks (such as drinking water before sleeping, forcing the subject to wake up) or techniques (such as noting down dreams immediately upon awaking) [12]. Hyperactivation of the stress system is apparently implicated with nightmares [13,14,15,16,17,18,19,20], yet the involvement of the hypothalamic–pituitary–adrenal (HPA) axis in dream recall and affect has been the subject of few studies. In this concise review, we present an overview of selected research on dream recall/affect concomitantly with the activity of the HPA axis. Two literature search strategies were applied, on PubMed/PubMedCentral and on Google Scholar. The first strategy included the following combination of keywords: (HPA OR ([hypothalamus OR pituitary] AND [adrenal OR cortisol])) AND (dream OR ([dream AND recall] OR [dream AND affect])). The second strategy included: cortisol AND (dream OR ([dream AND recall] OR [dream AND affect])). The PubMed/PubMedCentral strategy produced 19 articles (with duplicates) whereas the Google Scholar strategy produced 12,900 sources. Full-text versions of all the articles from PubMed/PubMedCentral and of the first 100 from Google Scholar were retrieved and critically appraised for key results, limitations, suitability, quality, results obtained and their interpretation and impact. Additional references were identified from the reference lists of the retrieved articles and were also appraised.

## 2. The HPA Axis

The HPA axis is considered to be the main effector of the human stress response; it is a complex feedback/homeostatic loop system [21]. Schematically, the hypothalamus secretes corticotrophin-releasing hormone (CRH) in a pulsatile circadian fashion, which prompts the corticotroph cells in the anterior pituitary to secrete adrenocorticotropic hormone (ACTH). The latter “whips” the adrenal cortex in producing steroid hormones: glucocorticoids (mainly cortisol, F) but also mineralocorticoids (mainly aldosterone, Aldo). There is also peripheral CRH production, which has a pro-inflammatory action. F exerts a negative effect on the secretion of CRH and ACTH. Besides CRH, arginine vasopressin (AVP) and interleukin-6 (IL-6) also induce ACTH secretion. Both F and Aldo in the circulation are protein bound [22] and inactive, whereas these hormones are free in saliva or the interstitial fluid. Enzymatic inactivation of F to cortisone occurs in the liver, as well as locally. Clearance of F is also crucial to its effects [21]. The glucocorticoid receptor (GR) has two isoforms (GRα and GRβ, respectively), while the mineralocorticoid receptor (MR) has an equal affinity for F and Aldo. In the cytoplasm, GR is attached to heat-shock proteins (HSPs); it has to be dissociated from HSPs to enter the nucleus. There are also other natural ligands for GRs. Additionally, GRs can be subjected to epigenetic modulation. The GR is widely expressed in the brain, whereas the MR is expressed mostly in the limbic region [23]. Contrary to primates, the expression of GR in the human hippocampus remains mostly stable with age [24]. The main endogenous end-product of the HPA axis is F, which, following a circadian rhythm, shows its highest concentrations 30–45 min after awakening (defining the so-called cortisol-awakening response; CAR) and its lowest levels in the evening; there are also ultradian rhythms [25]. The CAR usually shows small day-by-day fluctuations and is a handy index of the HPA axis function and a marker of mental health resilience.

## 3. The HPA Axis and Sleep

As stated above, the HPA axis shows circadian (and less clearly defined ultradian) rhythms. In normal sleepers, the nadir of ACTH and F is around midnight and their peak is upon awakening. During sleep, ACTH and F show secretion peaks in the “throughs” of the slow-wave sleep phases between REM sleep phases [26].

## 4. Dream Recall and the HPA Axis

Steroid receptor activation may have a biphasic effect on brain synaptic plasticity. Activation, at lower levels of the MR ligands, enhances long-term potentiation, whereas with activation, at higher levels of the GR ligands, long-term potentiation in inhibited, with a decrease in the excitability of the hippocampus [23]; the latter’s function is needed for “typical” dreaming and dream recall [27]. A role for the HPA and F in particular has been postulated regarding memory consolidation and subsequently for dream recall [28]. Specifically, F may have an effect on how emotions are experienced in the process of dream formation, via fragmentation of the phenomenological dream experience and via disruption of memory retrieval [26].

In a study with 188 women, those that reported frequent nightmares (*n* = 13) had a blunted CAR (with consistently lower CAR levels, assessed with saliva sampling) compared to the rest of the women, but only on working days [29]. Of note, between the two groups, no differences in F levels per se—upon awakening—were delineated [29]. In another study (*n* = 30) by the team of M. Schredl, a lower CAR (also in saliva samples) was associated with frequent/chronic nightmares, in contrast to acute nightmares, for which the hallmark was an elevated CAR, vis-à-vis nights with dreams of neutral content [30].

Patients with post-traumatic stress disorder (PTSD) report more nightmares and are characterized by lower F levels compared to healthy controls. Elegant mathematical models have been created for the description of the HPA axis functionality in such subjects; these models cannot account for all the variables (as described above) that define “active” F levels [31]. Low-dose hydrocortisone (rather than F, as stated in the guidelines) at 10 mg per day, either upon bedtime or split in two over the day, can be considered as treatment for PTSD-associated nightmares, but the data upon which this recommendation by the Standards of Practice Committee (SPC) of the American Academy of Sleep Medicine (AASM) is based are of low-grade quality [32].

Experimentally, auditory dream recall has been associated with lower (total) blood F levels [33]. In this study, participants were kept under anesthesia (vs. natural sleep) and subjected to auditory stimuli. The reported dreams appeared unrelated to the auditory stimuli and were mostly emotionally pleasant. This is in apparent contrast to studies (cited above) reporting an association between low F or CAR and nightmares [30,31].

In the laboratory setting, experimentally induced mental stress and the measured response in F may be enhanced in the luteal phase of the menstrual cycle [34]. In a study of 944 women with normal menstrual cycles, an association of pleasant dream content with the luteal phase was reported. We suggest that F in the luteal phase may selectively enhance dream amnesia, easing the recall of pleasant dreams [35].

## 5. Dream Recall in HPA Disease States

Chronic exposure to stress and subsequently protracted high levels of F or endogenous F increase (as in Cushing’s syndrome) lead to brain changes; among others, alterations in the hippocampus can include neuronal apoptosis and reduction in its size [36,37,38]. Proton magnetic resonance spectroscopy has shown metabolic disturbances in the prefrontal medial cortex of patients with Cushing’s syndrome [37]; this area is also implicated in dreaming and dream recall [39]. Subjects that perceive themselves as being stressed have higher CAR compared to healthy subjects. This index has also been associated with major depression [40]. Memory functions have been found to be impaired in subjects with chronically elevated F levels (patients with Cushing’s syndrome, major depression or schizophrenia) or even after a single dose of hydrocortisone [33]. In an old study, in one third of patients with Cushing’s syndrome, alterations in dream recall frequency (increased or decreased) and in dream content (with a shift towards more bizarre and vivid content) were reported [41].

Patients in intensive care units (ICU) show a protracted stress response, with modest elevation or high total F (attributed to lowering in protein binding/F clearance/free F elevation; these changes occur usually in the absence of high ACTH, which may be downregulated by high free F). Twenty-five percent of ICU patients are treated with glucocorticoids [23]. More than half of critically ill patients may (subsequently) report disturbed dreaming experiences and nightmares during long-term ICU hospitalization, as well as after hospital discharge [42,43,44,45].

In patients with adrenal insufficiency and low endogenous F levels, the scant relevant studies do not mention sleep/dream disturbances [46].

We have to note that, as in the preceding section, regarding published studies that hone in on nightmare frequency, the latter is not necessarily an adequate or thorough measure of negative dream affect [47]. Furthermore, the mechanisms through which waking state emotions influence dream emotions are still very poorly understood and recent data call into question the existence of a direct reflection of prevalent emotional valence of wake into that of the dream [48,49].

## 6. COVID-19 and Dreams

Memory impairment and insomnia, among others, have been reported as sequelae of COVID-19 infection; the relevant involvement of the HPA axis is highly likely, given the body’s stress system activation by the disease [21]. Interestingly, in the past, there was speculation that antibodies produced in patients with SARS-CoV-1 (causing SARS) could destroy ACTH, with subsequent blunting of the F response [50]. Nevertheless, overt hypocortisolism has not been verified either in patients with SARS or in patients with COVID-19 [50]. In critically ill COVID-19 patients, lower F has been measured compared to non-COVID-19 critically ill patients [51]. To the best of our knowledge, studies on the dreams of COVID-19 survivors have not been presented at the time of writing.

The COVID-19 global situation begets population-wide stress [52]. In a study from Spain (*n* = 45), 10–26% of the variance in perceived stress during confinement because of the COVID-19 situation was predicted by pre-confinement CAR and the Brief Resilient Coping Scale score [40]. Alterations in sleep onset, duration and quality have been reported in locked-down subjects in the current COVID-19 era [53], while in studies honed on dream content, one quarter of the participants reported an increase in nightmares (*n* = 811) compared to the pre-pandemic era [52] and aggressive dream content in women (*n* = 51) compared to men (*n* = 19) [54]. In web-based surveys in Italy during the COVID-19 pandemic (with *n* = 5988 and 1091), researchers found increased dream recall frequency, as well as nightmare frequency and bizarre dream content, compared to population-based pre-COVID-19 samples [55,56].

## 7. Conclusions

The necessity of dreams is still under investigation. Theorists of the psychoanalytic approach state that dreams are the window to the subconscious and the understanding of the human psyche and reflect our unconscious desires. It seems, however, that dreams play an important role in unifying our memory, as our brain processes the information of the day by eliminating irrelevant, unnecessary information and placing the important ones from short-term to long-term memory. The HPA axis is implicated in these memory processes and dream recall; however, the relevant research still presents a fragmented “scenario” in which a comprehensive hypothesis cannot yet be drawn. More questions begging for answers emerge. A state of protracted elevation in F may lead to a higher rate of dream recall of unpleasant dreams/nightmares, but the threshold of F for this is unknown. Is there a F cut-off in the context of this hormone’s normal circadian variation over which dream recall rate and content are affected? Are subtle perturbations of the HPA activity caused by daily wear and tear sufficient to have repercussions on dream recall and effect? Further studies, from varied populations, with larger samples (and higher power), using at least the CAR vis-à-vis dream recall/affect, will shed more light on the subject. Additionally, regarding dream content/nightmares, selective modulation of the GR and MR might have therapeutic potential [57].

## Data Availability

Not applicable.
